# Simultaneous ocular and osseous syphilis: a case report

**DOI:** 10.5935/0004-2749.20220043

**Published:** 2022

**Authors:** Juliana Albano de Guimarães, Marcelo Paccola

**Affiliations:** 1 Department of Ophthalmology and Otorhinolaryngology, Universidade Estadual de Campinas, Campinas, SP, Brazil; 2 Uveitis Service, Department of Ophthalmology and Otorhinolaryngology, Universidade Estadual de Campinas, Campinas, SP, Brazil

**Keywords:** Syphilis/complications, Neurosyphilis, Eye infections, bacterial, Uveitis, posterior, Chorioretinitis, Periostitis, Case report, Sífilis/complicações, Neurossífilis, Infecções oculares bacterianas, Uveíte posterior, Coriorretinite, Periostite, Relato de caso

## Abstract

Syphilis is a reemerging and potentially serious disease. Owing to its ubiquity and pleomorphism, it is called “the great imitator”. We report the case of a young woman with secondary syphilis who presented with bilateral acute syphilitic posterior placoid chorioretinopathy along with a syphilitic skull periostitis. A pachymeningeal enhancement was observed on magnetic resonance imaging, but we believe it was an extension of the bone process rather than a meningitis itself on the basis of the normal cerebrospinal fluid analysis results. Treatment with intravenous crystalline penicillin resulted in complete resolution of the signs, symptoms, and imaging findings. Secondary syphilis is the stage with the highest bacteremia and the highest transmissibility, presenting mainly with mucocutaneous disorders and, less frequently, with involvement of other organs. High suspicion and a pragmatic approach are essential to the diagnosis because this disease can affect several organs, as in the present case, in which the eyes, bones, and skin were affected.

## Abstract (pt)

A sífilis é uma doença reemergente e potencialmente grave. Por sua onipresença e pleomorfismo, é denominada “grande imitadora”. Relatamos caso de paciente jovem com sífilis secundária, que se apresentou com coriorretinopatia placóide sifilítica posterior aguda bilateral, simultaneamente a periostite craniana sifilítica. A despeito de realce paquimeníngeo observado na ressonância magnética, acreditamos que este tenha sido uma extensão do processo ósseo e não, uma meningite em si, uma vez que o exame do líquido cefalorraquidiano estava completamente normal. Tratamento com penicilina cristalina intravenosa resultou em completa resolução dos sinais, sintomas e achados de imagem. A sífilis secundária é o estágio de maior bacteremia e maior transmissibilidade da doença, apresentando-se principalmente com quadros mucocutâneos, mas também, menos frequentemente, com envolvimento de outros órgãos. Elevada suspeição e uma abordagem pragmática são necessárias para o diagnóstico, uma vez que essa doença pode afetar vários órgãos, como no caso relatado, em que foram acometidos olhos, ossos e pele.

## INTRODUCTION

Syphilis is a sexually transmitted systemic disease caused by the spirochete *Treponema pallidum*. Known as “the great imitator”, syphilis can present itself in several ways, reaching virtually any organ. In recent years, the increase in incidence, especially in the subgroup of patients with human immunodeficiency virus, has attracted attention to syphilis^(1)^.

We report a case of secondary syphilis with simultaneous ocular and osseous presentations in the form of bilateral acute syphilitic posterior placoid chorioretinopathy and syphilitic skull periostitis.

## CASE REPORT

A 45-year-old female patient presented with a bilateral rapidly progressive low visual acuity (VA) for five days, associated with severe headache, worse on the right side. A self-limited maculopapular rash was reported to have occurred three weeks earlier.

**Figure 1:**
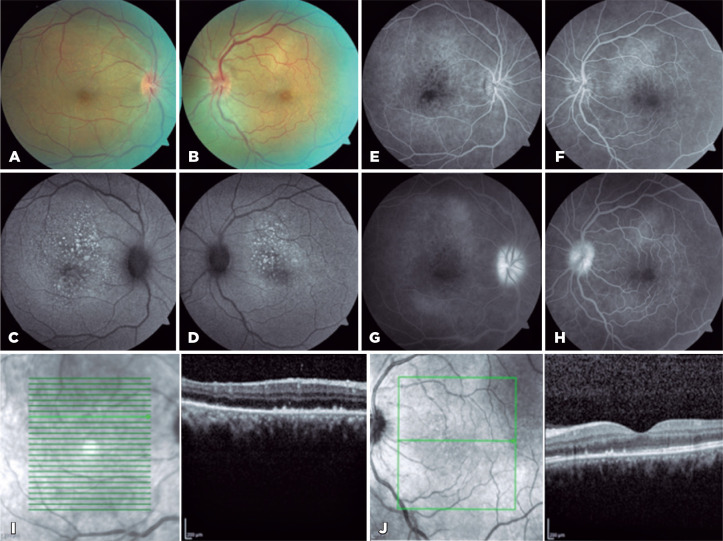
Posterior poles (A) of the right and (B) left eyes. Autofluorescence of the (C) right and (D) left eyes. Fluorescein angiograms of the (E) right and (F) left eyes in early stages, and (G) right and (H) left eyes in late stages. Optical coherence. tomography images of the (I) right and (J) left eyes.

VA was light perception in the right eye (oculus dexter [OD]) and 20/130 in the left eye (oculus sinister [OS]). A relative afferent pupillary defect was observed in the right eye. Anterior segment biomicroscopy and intraocular pressure measurement were normal. On fundoscopy, a yellowish, oval, placoid-shaped macular lesion was observed in both eyes (oculus uterque [OU]), in association with discrete vitreous cellularity and optic disk edema (Figure 1A and B).

Autofluorescence (AF) demonstrated a macular hyperautofluorescent granularity in OU (Figure 1C and D). Fluorescein angiography (FA) revealed staining of the optic disc and retina in late stages and an increased foveal avascular zone in OU (Figure 1E-H). Optical coherence tomography (OCT) revealed hyper-reflective nodularity along the retinal pigment epithelium (RPE) and ellipsoid disruptions in OU (Figure 1I and J).

The hypothesis of acute syphilitic posterior placoid chorioretinopathy was formulated, and laboratory workup revealed a Venereal Disease Research Laboratory (VDRL) test titration of 1:128, a positive fluorescent treponemal antibody absorption test (FTA-ABS) result, and a negative human immunodeficiency virus (HIV) serology. On the basis of the presence of ocular involvement and severe headache, the hypothesis of neurosyphilis was formulated, and magnetic resonance imaging (MRI) and cerebrospinal fluid (CSF) puncture were requested.

**Figure 2:**
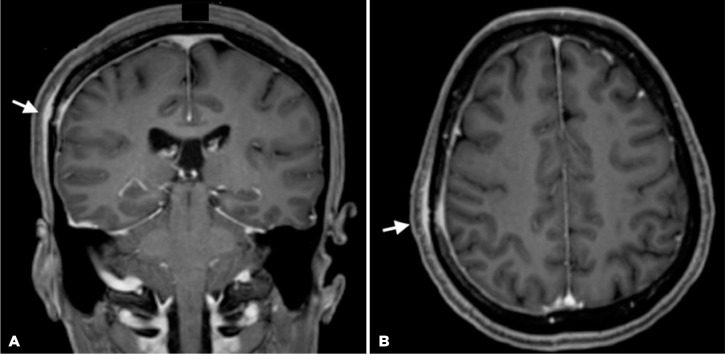
Magnetic resonance images of the (A) coronal and (B) axial frames.

MRI revealed a lesion with impregnation by contrast in the right frontoparietal skullcap and a smooth dural enhancement along the right convexity, suggestive of an inflammatory-infectious process (Figure 2). The cerebral parenchyma and optic nerve-sheath complexes demonstrated no changes. The CSF analysis revealed negative VDRL and FTA-ABS test results, no pleocytosis (5 leukocytes/mm^3^), and protein levels within the reference values (36,00 mg/dL). The opening pressure was 17 cm H_2_O.

**Figure 3:**
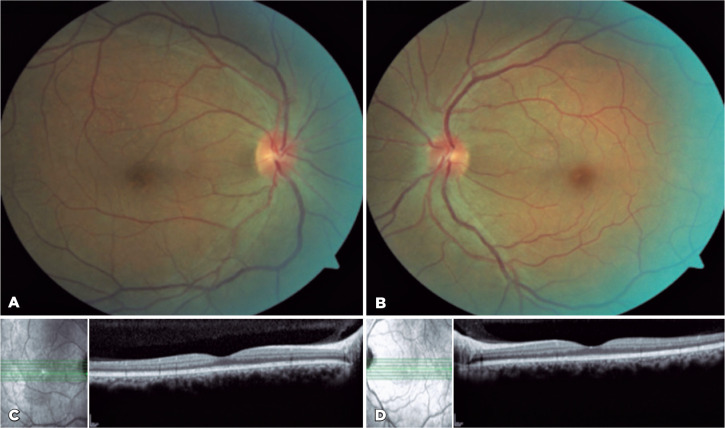
Fundus of the (A) right and (B) left eyes after treatment. Optical coherence tomography images of the (C) right and (D) left eyes after treatment.

**Figure 4:**
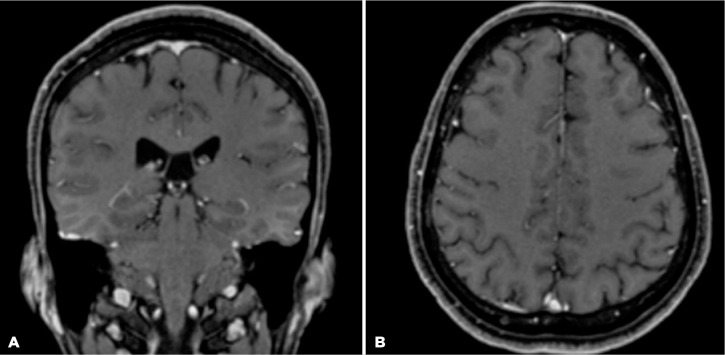
(A) Magnetic resonance images of the coronal and (B) axial frames after treatment.

Admission for intravenous administration of aqueous crystalline penicillin G for 14 days followed, with gradual improvement of signs and symptoms throughout hospitalization (Figure 3A and B). Two months after the treatment, the patient’s VA was 20/20 in OU, resolution of the RPE nodularities was observed on OCT (Figure 3C and D), and complete resolution of the dural enhancement and skull lesion were visualized on MRI (Figure 4). Follow-up in the ophthalmology and infectology departments were maintained.

## DISCUSSION

Syphilis is classically divided into stages (primary, se condary, latent, and tertiary), with secondary syphilis being the most florid stage of the disease, resulting from multiplication and dissemination of spirochetes. The manifestations are most often mucocutaneous, but other organs can be involved, such as the eyes, bones, and central nervous system (CNS)^(2)^.

The eye can be involved in the early and late stages, but most cases are found in secondary syphilis. Acute syphilitic posterior placoid chorioretinopathy is a suggestive and specific manifestation of syphilis and consists of an inflammatory condition that affects the external retina and internal choroid, manifesting as yellowish circular or oval placoid lesions in the posterior pole. Approximately 50% of the cases have a history of mucocutaneous involvement in the 12 months preceding the ocular presentation, and approximately 56.3% of patients have a bilateral condition. VA ranges from 20/20 to absence of light perception, and mild vitreous inflammation and optic disc edema can be observed^(3-5)^.

AF shows hyperautofluorescent lesions. On FA, hypofluorescent lesions can be observed in early stages, with progressive leakage and hyperfluorescence in late stages. On angiography with green indocyanine, hypocyanescent lesions can be found in early and late stages. OCT shows disruptions of the ellipsoid, accumulation of subretinal fluid in small amounts, and deposits at the level of the RPE. OCT angiography may reveal reduced blood flow at the level of the choriocapillaris, which can be correlated to an increased area of hypofluorescence by non-perfusion on FA, as observed in the reported case^(4-7)^.

Bone involvement is common in syphilis owing to the high affinity of the *T. pallidum* to osseous structures. The skull and long extremity bones are the most commonly involved bones, with symptoms consisting of headache and local pain, respectively^(8,9)^. Osseous involvement can be detected by imaging examination, and enhancement of the adjacent periosteum and dura-mater can be observed on MRI^(8)^.

The CNS can also be involved in early and late stages. One of the most frequent clinical patterns of CNS involvement in early syphilis consist of meningitis, which can be asymptomatic or present with headache, photophobia, nausea, vomiting, cranial nerve palsies, and seizures. Though more common in patients with HIV, symptomatic syphilitic meningitis can develop in immunocompetent individuals, especially when the titers of serum non-treponemal markers are high^(1,5,10)^. The diagnosis of neurosyphilis is based on CSF analysis results. A positive VDRL or FTA-ABS test result establishes the diagnosis, but while the first test has low sensitivity (30-90%) in the CSF, the second test has a sensitivity of >90%. Furthermore, pleocytosis and increased protein levels are expected^(11)^.

In face of optic disc edema and dural enhancement on MRI, one could have raised the hypothesis of pachymeningitis as a differential diagnosis in the reported case. However, a normal opening pressure, negative FTA-ABS test result, negative VDRL, absence of pleocytosis, and normal protein levels in the CSF made the diagnosis of pachymeningitis unlikely.

Neurosyphilis can occur concomitantly or as an extension of ocular or osseous syphilis, considering its anatomical proximity. Ocular syphilis is included in the spectrum of neurosyphilis, being treated with intravenous aqueous crystalline penicillin G in the same way as neurosyphilis. Osseous syphilis, on the other hand, may be treated with intramuscular penicillin G benzathine, as long as neurosyphilis has been ruled out. However, in the presence of dural enhancement on MRI, it may be advisable to treat osseous syphilis with intravenous aqueous crystalline penicillin G, even in the absence of CSF alterations^(9,12)^.

The overall and visual prognosis is generally good when adequate treatment is established. As in the reported case, VA is improved in most patients, with a mean final VA of 20/25^(4)^. The complete resolution of signs and symptoms demonstrated in this report endorses the importance of diagnosing and treating syphilis, which is a potentially serious but treatable disease.
